# Final Scenes: Death Literacy Through Devised Theater

**DOI:** 10.1093/geroni/igaf122.2100

**Published:** 2025-12-31

**Authors:** Carol Weisse

**Affiliations:** Union College, Schenectady, New York, United States

## Abstract

Most people wish to die in the comfort of home, but few possess a realistic understanding of the challenges patients and families face when death occurs outside of a medical setting. Through well-established research practice partnerships with several hospice residential care homes, we have curated an extensive collection of caregiver narratives describing the day-to-day end-of-life journeys of over 350 deceased hospice patients. These care narratives provide a special lens to the dying experience in a raw, holistic, detailed manner, offering a rare opportunity to examine and interrogate the death and dying process when it occurs in a home setting. This presentation will describe a community-based participatory artistic endeavor through which an interdisciplinary team of students, faculty, local theater experts, and community caregivers examined hospice caregiver narratives with the goal of developing a series of ten, 10-minute plays capturing the diverse final journeys of terminally ill patients and those caring for them. This presentation will describe the collective creation process of devised theater and how a team-based approach for devising plays about death and dying provides an opportunity for joint examination of the challenges associated with accessing and receiving home-based hospice care. There will be a discussion of the use of caregiver narratives in developing public performances aimed at improving death literacy, and the presentation will close with a brief reading of a devised play and the play’s accompanying discussion questions developed for use in facilitating community conversations about death and dying.

